# Historical perspective: Revisiting the St. Lucia Project, a multi-year comparison trial of schistosomiasis control strategies

**DOI:** 10.1371/journal.pntd.0006223

**Published:** 2018-01-31

**Authors:** Julianne A. Ivy, Charles H. King, Joseph A. Cook, Daniel G. Colley

**Affiliations:** 1 Center for Global Health and Diseases, School of Medicine, Case Western Reserve University, Cleveland, Ohio, United States of America; 2 Schistosomiasis Consortium for Operational Research and Evaluation, University of Georgia, Athens, Georgia, United States of America; 3 Gillings School for Global Public Health, University of North Carolina, Chapel Hill, North Carolina, United States of America; Australian National University, AUSTRALIA

## Introduction

In 1965, the government of St. Lucia and the Rockefeller Foundation undertook what became a sixteen-year project to determine the optimal strategy for controlling locally-endemic schistosomiasis mansoni. Many of the world’s leading researchers on schistosomiasis control participated in the project, including experts in epidemiology, snail ecology, water and sanitation, social mobilization, clinical trials, immunology, and health economics. In the process, they brought infection levels in the new island nation to an impressive and steady low. Now fifty years later, the island has maintained its control of the parasite and may be on the cusp of achieving national *Schistosoma mansoni* elimination status. There are many other countries still fighting endemic schistosomiasis, and for them, achieving elimination might seem an elusive goal. However, the research evidence from the St. Lucia project, as documented in its nearly 140 research publications and as summarized in book form by Peter Jordan in 1985 [1], provides many lessons that can be applied to countries battling *Schistosoma* transmission today (Box 1). For readers interested in the details of study design and the full results of the trials, we have included supplemental S1 and S2 Files at the end of this review to provide searchable listings of the many scientific reports published by the Project.

**Box 1. Significant facets of the St. Lucia study**

- The St. Lucia study, performed during 1965–1981, was the first large-scale concurrent comparison study of drug-based and environmental approaches to control of *S*. *mansoni* prevalence and transmission
- The study compared targeted chemotherapy to long term intermediate-host snail control and to implementation of water and sanitation measures
- Crossover trials at the end of the study yield us our best estimates for the impact of combined intervention
- It is important to revisit this extensive research experience in light of the upcoming revision of WHO guidelines for schistosomiasis control

### The 1965–1981 St. Lucia study was the first large-scale concurrent comparison trial of strategies for control of *S*. *mansoni*

**Fig 1:**
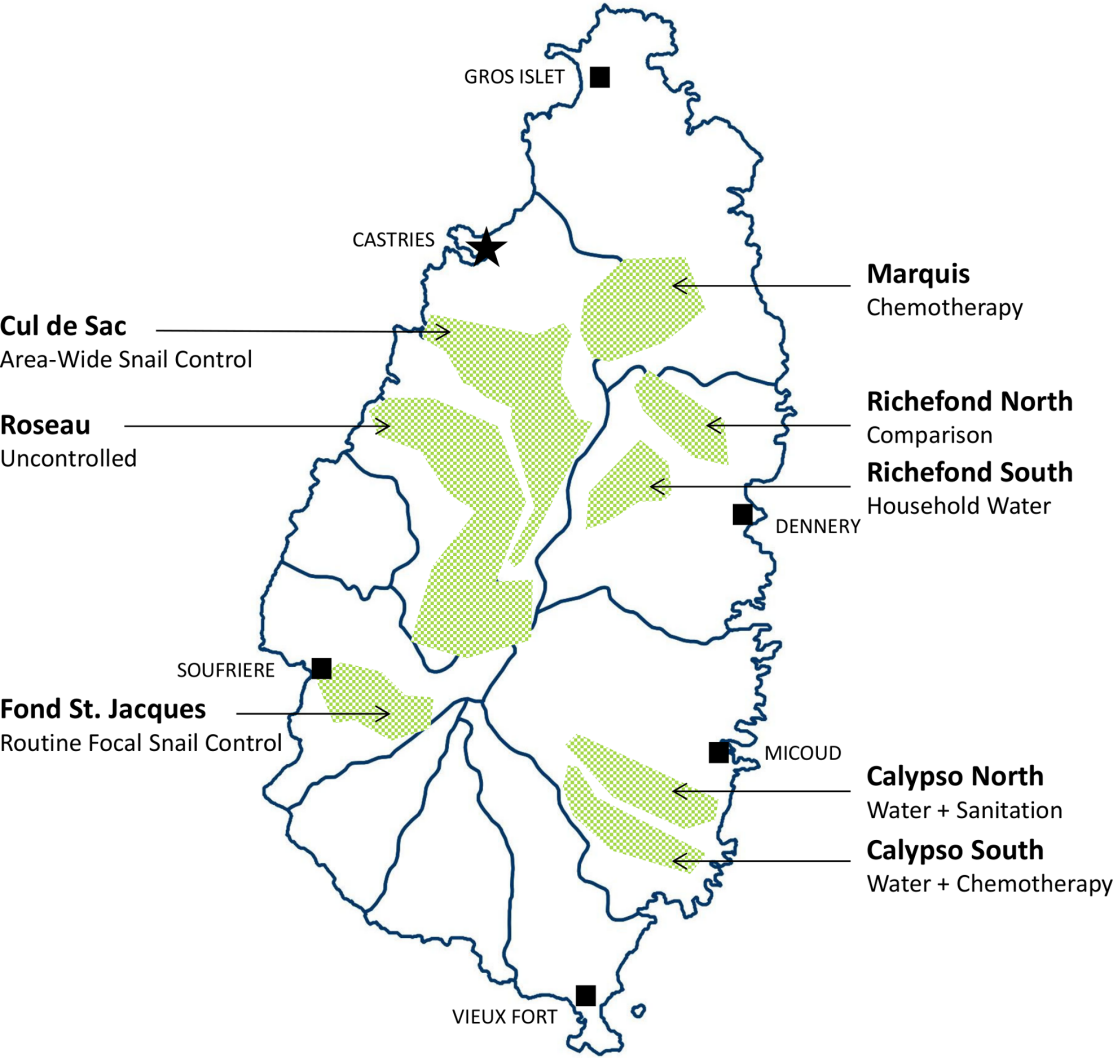
Sketch map of St. Lucia Island, indicating valleys selected for intervention assignments in the 1965–1981 comparison trial of *S*. *mansoni* control strategies. Shaded areas indicate the separate valley locations where the different study interventions were tested during the course of the St. Lucia Project. Labels indicate the names of the selected valleys and which interventions were performed at each location. Map created with QGIS v.2.18.10 software (http://www.qgis.org) using a country base map provided by the GADM database of Global Administrative Areas (http://www.gadm.org).

In 1965, the St. Lucia government and the Rockefeller Foundation founded the “Research and Control Department of the Ministry of Health” as a focus for their collaboration on schistosomiasis mansoni control. Their first objective was to get the island’s increasing levels of infection under control. Pre-intervention studies in the high-transmission areas of each targeted valley found a median *S*. *mansoni* prevalence of 45% in children [1, p.270], with age-group prevalences reaching up to 91% in individual areas [1, p.53]. Serious illness from schistosomiasis was not uncommon, with a number of children under age 14 having egg counts over 1000, and many more with enlarged livers or spleens [2]. The researchers recognized that while a number of schistosomiasis control methods had been individually tested in trials previously performed in other countries, none had performed head-to-head trials to determine which single method was likely to be most successful in controlling disease or in preventing transmission [1, p.6]. The mountainous terrain of St. Lucia could allow researchers to test individual control methods in relatively isolated valleys (Fig 1), so the newly-constituted Department designed a large-scale, concurrent comparison study of drug-treatment vs. intermediate-host snail control vs. improvement of water supplies, which was the first study of its kind.

The three main control methods tested in St. Lucia were: (i) targeted chemotherapy, (ii) chemical and environmental snail control (both area-wide and then focal), and (iii) installation of household water supplies with eventual provision of community showers, laundry units, and recreational water facilities. Each strategy presented its own specific set of challenges and technical demands, as noted in Table 1. Installation and maintenance of water supplies proved to be generally the most demanding intervention in terms of supervision, and chemotherapy the least. Snail control required the least amount of direct participation from the community.

**Table 1:** Comparison of requirements and effects of control strategies tested in the St. Lucia Project (adapted from reference [1]).

	Snail control	Chemotherapy	Clean water
**Required pre-implementation investigations**	Transmission patterns	Case detection / prevalence surveys	Location of clean water sources
**Required personnel**	Public health workers	Technicians, Paramedics	Engineers, Artisans
Required supervision, initially*	+ + +	+ +	+ + + +
Required supervision, maintenance-phase*	+ +	+	+ +
Required community participation and health education*	+	+ + + +	+ + + +
**Required materials**	Chemicals	Drugs, Diagnostic tools	Pipes, fitting, pumps, tanks, electricity, etc.
**Onset of Effect**	Slow	Rapid	Slow
**Population protected**	All those using treated water bodies	Those treated; untreated may become less exposed to infection	Those using water supplies
**Other benefits**	Minimal	Patients cured, Pathology reversed	Improved overall health, Social benefits
**Impact of infected migrants**	Little impact	Significant impact (i.e. undermined by immigration)	Little impact

* Key: + least; + + + + most

### Comparing targeted chemotherapy to long-term host snail control and to provision of household water supplies

#### Drug treatment

Targeted chemotherapy trials were organized in St. Lucia’s Marquis Valley in the northeastern sector of the island (Fig 1). Annually from 1973–1976, all children and adults who tested positive for *S*. *mansoni* (by the sedimentation stool exam technique) were given treatment with intramuscular hycanthone (1973 and 1974) or oral oxamniquine (1975 and 1976) [3]. This chemotherapy intervention, given alone, resulted in the largest reductions in local prevalence and yearly incidence, as compared to locations having snail control alone or piped water installation alone. Specifically, incidence of *S*. *mansoni* in high transmission areas was reduced from 22% to 4% per annum among young children under the age of 10, and existing prevalence was reduced from 41% to 4% [1, p.270]. Drug treatment was the fastest-acting intervention, with the most rewarding short-term results [4]. However, a mild increase in *S*. *mansoni* prevalence occurred between the third and fourth treatments, and this foreshadowed a later resurgence of transmission in some sectors of the valley [1, p.103].

#### Water and sanitation

In the southwest part of Richefond Valley, piped water was installed and supplied to 386 individual household taps, serving around 2,000 people; then laundry units, showers, and recreational pools were subsequently provided for the community [5]. A health education program was also implemented in this valley when it became clear that not everyone reduced their contact with streams and rivers after provision of the new water supplies [1, p.212]. Compared to snail control and to chemotherapy, provision of clean water (alone) had the greatest effect on the mean egg output of infected children, yielding a 50% overall reduction in their infection intensity [1, p.270]. Incidence per annum of new *S*. *mansoni* infection also decreased from 23% to 13% among children under 10, and overall prevalence decreased from 56% to 38% [1, p.270]. An additional trial involving installation of water-seal latrines was undertaken in the northern part of Calypso, but due in part to irregular water supplies during a period of drought, this additional intervention did not end up significantly affecting *Schistosoma* transmission [1, p.255].

#### Snail control

The primary snail control trials took place in Cul de Sac Valley. After an initial period of three years to establish baseline data and to map breeding sites, the Department implemented area-wide mollusciciding with niclosamide from 1970 to 1975 [1, pp.118/131]. This involved initially treating all known snail habitats, and then routinely re-surveying these habitats and treating only those where snails were found. This was performed at 2-week intervals for streams and marshes and at 3 to 4-week intervals for banana drains [1, pp.136/165]. This aggressive, wide-coverage campaign brought human incidence per annum of *S*. *mansoni* down from 23% to 6% and prevalence from 45% to 24% among children in high-transmission areas of the Cul de Sac Valley [1, p.270]

**Fig 2:**
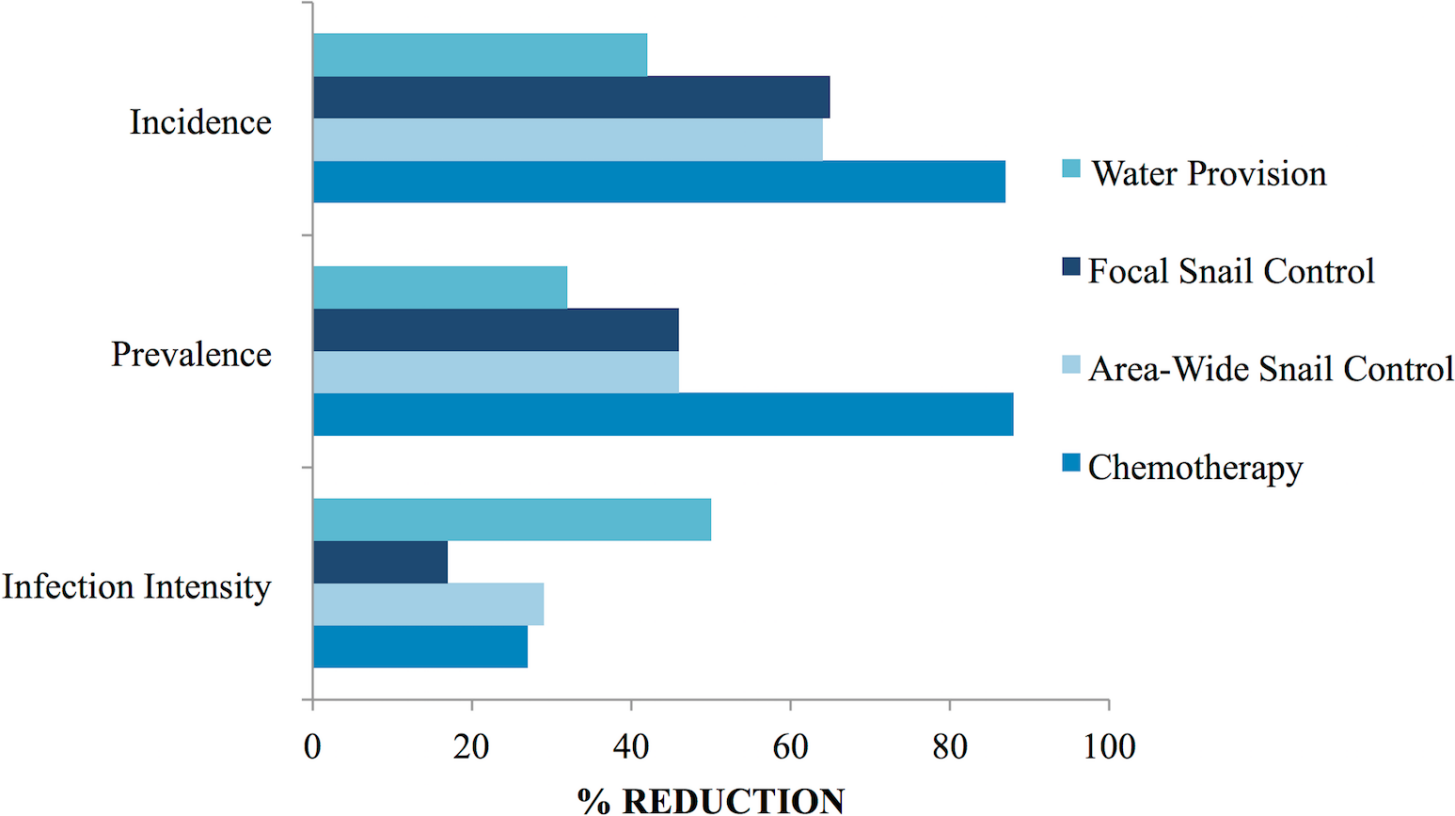
Comparison of the impact of individual control measures implemented during the St. Lucia study. The effects of provision of water, of focal and area-wide snail control via mollusciciding, and of targeted chemotherapy in reducing *S*. *mansoni* incidence per annum, prevalence, and mean infection intensity (for those with egg-positive stools). Relative reductions over the initial 3–5 year course of each intervention are shown (as percentages) indicated by the horizontal bars. Data from [1, pp. 270–271].

Even with this success, the investigators realized that the costs and time demands of such intensive intervention would be prohibitively high for many resource-poor countries where schistosomiasis is endemic. As a result, they chose to test a new, less costly alternative, which involved using only focal snail control at specific transmission sites [6]. For this trial, they chose the Fond St. Jacques region of St. Lucia. In this second test, the researchers identified 12 sections of stream where infected *Biomphalaria*. *glabrata* had been found and where humans had frequent contact with the water [1, p.181]. These were selected as the most likely sites of transmission and thus were made the sole locations for monthly niclosamide treatments between 1976 and 1980, regardless of snail presence [6]. In high transmission areas of this valley, yearly incidence dropped from 15% to 8% and prevalence from 43% to 22% among children under ten years old [1, p.270]. Researchers concluded that, although more limited in scope, routine focal snail control was sufficient for decreasing infection levels, and they recommended continuing this intervention at 6-week intervals for the foreseeable future [7–10,] (Fig 2).

In 1978, Project researchers began a trial of supplementary biological control of the intermediate host *B*. *glabrata* snails by means of introducing non-native competitor snails such as *Helisoma duryi* and *Melanoides tuberculata* to local aquatic habitats, particularly to known transmission sites [11, 12]. Post-Project snail surveys in the late 1980s, the 1990s, and in recent years indicate that *M*. *tuberculata* has become the most common freshwater snail in St. Lucia [12]. There is now an absence or very low density of *B*. *glabrata* in many former *S*. *mansoni* transmission sites, suggesting a major shift in the snail ecology of the island [12]. This environmental change may explain, in part, the very low numbers of human *S*. *mansoni* infections now reported by island health service workers.

### Follow-on crossover trials that give us our best estimates for the impact of combining interventions

Anti-schistosomal chemotherapy was arguably the most successful single intervention during the initial phase of the trials. However, after the four annual chemotherapy campaigns were completed in the Marquis Valley (1973–76), the yearly incidence of *S*. *mansoni* infection rose again in Fond Assau and Talvern areas of the valley, without a clear explanation [1, p.106]. Three years after the final treatment, their incidence had returned nearly to pre-intervention levels, and infected snails were found in streams around the Marquis Valley [1, p.115; 7]. In addition to offering chemotherapy to residents again in 1979 and 1980, the Department responded by adding focal snail control to this site in March of 1980 [1, pp.95/113]. In the last 12 months of the St. Lucia project, no more infected snails were found in Marquis Valley, suggesting that the combined approach was significantly more successful in achieving interruption of transmission [1, p.115].

In South Richefond, schistosomiasis declined after installation of the new, household level water supplies. As the project progressed, and oral oxamniquine made anti-schistosomal treatment more accessible, chemotherapy was offered to any local Richefond residents who were still infected between 1975 and 1977 [1, p.199]. The household water plus treatment approach was seen by the Department as a way of strengthening the effects of chemotherapy because the household water-related reductions in yearly incidence of *S*. *mansoni* infection meant that people were less likely to become immediately re-infected after treatment [1, p.199]. Interestingly, a temporary resurgence in infections was seen when community members, interpreting their reported low incidence as meaning their rivers were safe, returned to the still-infested waters and yearly incidence again increased to pre-control levels [1, p.199]. This added chemotherapy campaign was carried out for 3 years, and afterwards, no further intervention was done other than maintaining the relatively costly water systems [1, pp.199/207]. By the end of the study in 1981, following this combination of interventions, annual incidence among children was at only 1% and prevalence was at 9% [1, p.271]

In Cul de Sac Valley, after the initial 1970–75 area-wide snail control program, the research team launched two further years of focal snail surveillance and control [1, p.152; 8], combined with targeted chemotherapy [1, p.154; 9]. This combined intervention was successful and further reduced prevalence from 24% to 6% [1, p.271]. Subsequently, the Department chose 12 streams and 26 banana drains (out of 145 initial sites) to undergo routine mollusciciding every four weeks regardless of observed snail levels [10]. This regimen continued for the last four years of the St. Lucia project and was sufficient to prevent any resurgence of infection [10].

### Importance of reviewing these outcomes in light of upcoming plans for revisions to WHO guidelines for schistosomiasis control

With good reason, mass drug administration is now the mainstay of schistosomiasis morbidity control. But when chemotherapy fails to prevent reinfection, alternative control methods must be considered to interrupt transmission. The Marquis Valley chemotherapy trial was significantly enhanced when supplemented with snail control, and Cul de Sac and South Richefond obtained impressive results by supplementing snail control or WASH-related measures with chemotherapy. The St. Lucia study was not a randomized trial, and very local factors may have biased some of its findings. However, its extensive observational data on the concurrent implementation of very different approaches to schistosomiasis control offers a very strong base of evidence for modern-day decision-making regarding potential elimination strategies. The project’s results are not fully applicable to every country fighting schistosomiasis, particularly given St. Lucia’s island ecology and the fact that the study only dealt with *S*. *mansoni*. Nevertheless, the island has significantly minimized *Schistosoma* infections, and the results of the multiple approaches that it undertook back then deserve a close second look today.

## Supporting information

S1 File Listing of St. Lucia Project research papers 1963–1993. This document file (.docx) provides an alphabetical listing, by author, of 133 published research papers related to the St. Lucia Project.
(DOCX)

S2 File St. Lucia Project research papers in EndNote format (.enlx). This searchable database file contains information on 133 published research papers related to the St. Lucia Project and their related citation meta-data in EndNote file format (.enl plus supplemental files in a compressed.zip file).
(ZIP)
