# Supplementary material for: Historical perspective: Revisiting the St. Lucia Project, a multi-year comparison trial of schistosomiasis control strategies
Source: PLoS Negl Trop Dis. 2018 Jan 31;12(1):e0006223. doi: 10.1371/journal.pntd.0006223 (PMC5791936; doi:10.1371/journal.pntd.0006223)
Supplement: S1 File — This document file (.docx) provides an alphabetical listing, by author, of 133 published research papers related to the St. Lucia Project. (DOCX) [file pntd.0006223.s001.docx]

**Publications on the work of the Research and Control Department, St. Lucia**

[1-133]

1. Barnish G. Evaluation of chemotherapy in the control of *Schistosoma mansoni* in Marquis Valley, Saint Lucia. II. Biological results. Am J Trop Med Hyg. 1982;31(1):111-115.

2. Barnish G. The freshwater shrimps of St. Lucia, West Indies (Decapoda: Natantia). Crustaceana. 1984;47:314-320.

3. Barnish G, Christie JD, Prentice MA. *Schistosoma mansoni* control in Cul de Sac Valley, Saint Lucia. I. A two-year focal surveillance-mollusciciding programme for the control of *Biomphalaria glabrata*. Trans R Soc Trop Med Hyg. 1980;74(4):488-492.

4. Barnish G, Jordan P, Bartholomew RK, Grist E. Routine focal mollusciciding after chemotherapy to control *Schistosoma mansoni* in Cul de Sac valley, Saint Lucia. Trans R Soc Trop Med Hyg. 1982;76(5):602-609.

5. Barnish G, Prentice MA. Lack of resistance of the snail *Biomphalaria glabrata* after nine years of exposure to Bayluscide. Trans R Soc Trop Med Hyg. 1981;75(1):106-107.

6. Barnish G, Prentice MA. Predation of the snail *Biomphalaria glabrata* by freshwater shrimps in St. Lucia, West Indies. Ann Trop Med Parasitol. 1982;76(1):117-120.

7. Barnish G, Prentice MA, Harris S. *Fasciola hepatica* in St Lucia, West Indies. Br Vet J. 1980;136(3):299-300.

8. Barnish G, Sturrock RF. Letter: Aerial application of a molluscicide to a marsh. Trans R Soc Trop Med Hyg. 1973;67(4):610-611.

9. Barsoum IS, Todd CW, Habib M, El Alamy MA, Colley DG. The effects of indomethacin on in vitro peripheral blood mononuclear cell reactivity in human schistosomiasis. Parasite Immunol. 1983;5(5):441-447.

10. Bartholomew RK, Goddard MJ. Quality control in laboratory investigations on *Schistosoma mansoni* on St Lucia, West Indies: a staff assessment scheme. Bull World Health Organ. 1978;56(2):309-312.

11. Bartholomew RK, Jordan P. Quality control in laboratory investigations on *Schistosoma mansoni* in St. Lucia, West Indies. Maintenance of artificial prevalence as a laboratory aid. Bull World Health Organ. 1978;56(4):655-656.

12. Bartholomew RK, Peters PAS, Jordan P. Schistosomiasis in St. Lucia and Kenyan communities - a comparative study using the Kato stool examination technique. Annals of Tropical Medicine and Parasitology. 1981;75:401-405.

13. Basch PF, Sturrock RF. Life history of *Ribeiroia marini* (Faust and Hoffman, 1934) comb. N. (Trematoda: Cathaemasiidae). Journal of Parasitology. 1969;55:1180-1184.

14. Celestin HN. Schistosomiasis control programme in St. Lucia: results of education scheme. Int J Hlth Educ. 1976;19:248-259.

15. Christie JD, Edward J, Goolaman K, James BO, Simon J, Dugat PS, et al. Interactions between St. Lucian *Biomphalaria glabrata* and *Helisoma duryi,* a possible competitor snail, in a semi-natural habitat. Acta Trop. 1981;38(4):395-417.

16. Christie JD, Prentice MA. The relationship between numbers of *Schistosoma mansoni* daughter sporocysts and miracidia. Ann Trop Med Parasitol. 1978;72:197-198.

17. Christie JD, Prentice MA, Upatham ES, Barnish G. Laboratory and field trials of a slow-release copper molluscicide in St. Lucia. Am J Trop Med Hyg. 1978;27(3):616-622.

18. Christie JD, Upatham ES. Control of *Schistosoma mansoni* transmission by chemotherapy in St. Lucia. II. Biological results. Am J Trop Med Hyg. 1977;26(5 Pt 1):894-898.

19. Colley DG, Cook JA, Freeman GL, Jr., Bartholomew RK, Jordan P. Immune responses during human schistosomiasis mansoni. I. In vitro lymphocyte blastogenic responses to heterogeneous antigenic preparations from schistosome eggs, worms and cercariae. Int Arch Allergy Appl Immunol. 1977;53(5):420-433.

20. Colley DG, Hieny SE, Bartholomew RK, Cook JA. Immune responses during human schistosomiasis mansoni. III. Regulatory effect of patient sera on human lymphocyte blastogenic responses to schistosome antigen preparations. Am J Trop Med Hyg. 1977;26(5 Pt 1):917-925.

21. Colley DG, Lewis FA, Goodgame RW. Immune responses during human schistosomiasis mansoni. IV. Induction of suppressor cell activity by schistosome antigen preparations and concanavalin A. J Immunol. 1978;120(4):1225-1232.

22. Colley DG, Todd CW, Lewis FA, Goodgame RW. Immune responses during human schistosomiasis mansoni. VI. In vitro nonspecific suppression of phytohemagglutinin responsiveness induced by exposure to certain schistosomal preparations. J Immunol. 1979;122(4):1447-1453.

23. Cook JA, Baker ST, Warren KS, Jordan P. A controlled study of morbidity of schistosomiasis mansoni in St. Lucian children, based on quantitative egg excretion. Am J Trop Med Hyg. 1974;23(4):625-633.

24. Cook JA, Jordan P. Excretion of *Schistosoma mansoni* ova in the urine. Trans R Soc Trop Med Hyg. 1970;64(5):793-794.

25. Cook JA, Jordan P. Clinical trial of hycanthone in schistosomiasis mansoni in St. Lucia. Am J Trop Med Hyg. 1971;20(1):84-88.

26. Cook JA, Jordan P. Clinical trials of hycanthone in schistosomiasis mansoni in St. Lucia. Egypt J Bilharz. 1974;1(2):197-202.

27. Cook JA, Jordan P. Absence of liver toxicity in 2723 patients treated with hycanthone in St. Lucia. Ann Trop Med Parasitol. 1976;70(1):109-111.

28. Cook JA, Jordan P, Armitage P. Hycanthone dose-response in treatment of schistosomiasis mansoni in St. Lucia. Am J Trop Med Hyg. 1976;25(4):602-607.

29. Cook JA, Jordan P, Bartholomew RK. Control of *Schistosoma mansoni* transmission by chemotherapy in St. Lucia. I. Results in man. Am J Trop Med Hyg. 1977;26(5 Pt 1):887-893.

30. Cook JA, Jordan P, Woodstock L, Pilgrim V. A controlled trial of hycanthone and placebo in schistosomiasis mansoni in St. Lucia. Ann Trop Med Parasitol. 1977;71(2):197-202.

31. Cook JA, Kellermeyer WF, Warren KS, Kellermeyer RM. Sickle cell hemoglobinopathy and *Schistosoma mansoni* infection. Ann Trop Med Parasitol. 1972;66:197-202.

32. Cook JA, Sturrock RF, Barnish G. An allergic skin reaction to a new formulation of the molluscicide clonitralide (Bayluscide). Trans R Soc Trop Med Hyg. 1972;66(6):954-955.

33. Cook JA, Warren KS, Jordan P. Passive transfer of immunity in human schistosomiasis mansoni: attempt to prevent infection by repeated injections of hyperimmune antischistosome gamma globulin. Trans R Soc Trop Med Hyg. 1972;66(5):777-780.

34. Cook JA, Woodstock L, Jordan P. Immunological studies in *Schistosoma mansoni* infection in St. Lucia. Ann Trop Med Parasitol. 1972;66(3):369-373.

35. Cook JA, Woodstock L, Jordan P. Two-year follow-up of hycanthone-treated schistosomiasis mansoni patients in St. Lucia. Am J Trop Med Hyg. 1974;23(5):910-914.

36. Cottrell BJ, Humber D, Sturrock RF. An immunosuppressive factor in the serum of patients with schistosomiasis. Trans R Soc Trop Med Hyg. 1980;74(3):415-416.

37. Dalton PR. A socioecological approach to the control of *Schistosoma mansoni* in St Lucia. Bull World Health Organ. 1976;54(5):587-595.

38. Evans AS, Cook JA, Kapikian AZ, Nankervis G, Smith AL, West B. A serological survey of St Lucia. Int J Epidemiol. 1979;8(4):327-332.

39. Goddard MJ, Jordan P. On the longevity of *Schistosoma mansoni* in man on St. Lucia, West Indies. Trans R Soc Trop Med Hyg. 1980;74(2):185-191.

40. Goodgame RW, Bartholomew RK. Lack of association of hepatosplenic schistosomiasis and alpha-1-antitrypsin deficiency. Am J Trop Med Hyg. 1978;27(4):779-781.

41. Goodgame RW, Colley DG, Draper CC, Lewis FA, McLaren ML, Pelley RP. Humoral immune responses in human hepatosplenic schistosomiasis mansoni. Am J Trop Med Hyg. 1978;27(6):1174-1180.

42. Henry FJ. Environmental sanitation infection and nutritional status of infants in rural St. Lucia, West Indies. Trans R Soc Trop Med Hyg. 1981;75(4):507-513.

43. Howell SB, Cook JA. Treatment of schistosomiasis mansoni with hycanthone in glucose-6-phosphate dehydrogenase deficiency in St. Lucia. Trans R Soc Trop Med Hyg. 1971;65(3):331-333.

44. Jordan P. Schistosomiasis--research to control. Am J Trop Med Hyg. 1977;26(5 Pt 1):877-886.

45. Jordan P. Oxamniquine and fever. Brit Med Bull. 1978;2:1366.

46. Jordan P, Barnish G, Bartholomew RK, Grist E, Christie JD. Evaluation of an experimental mollusciciding programme to control *Schistosoma mansoni* transmission in St Lucia. Bull World Health Organ. 1978;56(1):139-146.

47. Jordan P, Bartholomew RK, Grist E, Auguste E. Evaluation of chemotherapy in the control of *Schistosoma mansoni* in Marquis Valley, Saint Lucia. I. Results in humans. Am J Trop Med Hyg. 1982;31(1):103-110.

48. Jordan P, Bartholomew RK, Peters PAS. A community study of *Schistosoma mansoni* egg excretion assessed by the Bell and modified Kato technique. Annals of Tropical Medicine and Parasitology. 1981;75:35-40.

49. Jordan P, Bartholomew RK, Unrau GO, Upatham ES, Grist E, Christie JD. Further observations from St Lucia on control of *Schistosoma mansoni* transmission by provision of domestic water supplies. Bull World Health Organ. 1978;56(6):965-973.

50. Jordan P, Christie JD, Unrau GO. Schistosomiasis transmission with particular reference to possible ecological and biological methods of control. A review. Acta Trop. 1980;37(2):95-135.

51. Jordan P, Cook JA, Bartholomew RK, Grist E, Auguste E. *Schistosoma mansoni* control in Cul de Sac Valley, Saint Lucia. II. Chemotherapy as a supplement to a focal mollusciciding programme. Trans R Soc Trop Med Hyg. 1980;74(4):493-500.

52. Jordan P, Unrau GO. Simple water supplies to reduce schistosomiasis. Trop Doct. 1978;8(1):13-18. doi: 10.1177/004947557800800107.

53. Jordan P, Unrau GO, Bartholomew RK, Cook JA, Grist E. Value of individual household water supplies in the maintenance phase of a schistosomiasis control programme in Saint-Lucia, after chemotherapy. Bull World Health Organ. 1982;60(4):583-588.

54. Jordan P, Woodstock L, Cook JA. Preliminary parasitological results of a pilot mollusciciding campaign to control transmission of *Schistosoma mansoni* in St Lucia. Bull World Health Organ. 1976;54(3):295-302.

55. Jordan P, Woodstock L, Unrau GO, Cook JA. Control of *Schistosoma mansoni* transmission by provision of domestic water supplies. A preliminary report of a study in St Lucia. Bull World Health Organ. 1975;52(1):9-20.

56. Kellermeyer RW, Warren KS, Waldmann TS, Cook JA, Jordon P. Concentration of serum immunoglobulins in St. Lucians with schistosomiasis mansoni compared with matched uninfected St. Vincentians. J Infect Dis. 1973;127(5):557-562.

57. Lees RE. Lucanthone hydrochloride in the treatment of Schistosoma mansoni infection. Trans R Soc Trop Med Hyg. 1966;60(2):233-236.

58. Lees RE. Trial of an enteric-coated preparation of lucanthone hydrochloride in Schistosoma mansoni infection. Trans R Soc Trop Med Hyg. 1967;61(6):806-811.

59. Lees RE. Suppressive treatment of schistosomiasis mansoni with spaced doses of lucanthone hydrochloride. Trans R Soc Trop Med Hyg. 1968;62(6):782-785.

60. Lees RE. Symptoms and clinical and laboratory findings in 123 cases of schistosomiasis mansoni in St. Lucia. J Trop Med Hyg. 1968;71(2):40-43.

61. Lees RE. Regression of hepatosplenomegaly in schistosomiasis mansoni with steroid therapy. Trans R Soc Trop Med Hyg. 1968;62:296-297.

62. Lees RE, Jordan P. Transplacental transfer of antibodies to *Schistosoma mansoni* and their persistence in infants. Trans R Soc Trop Med Hyg. 1968;62(5):630-631.

63. Lewis FA, Sher A, Colley DG. Failure of plasma from human schistosomiasis mansoni patients to protect mice from *Schistosoma manson*i cercarial challenge. Am J Trop Med Hyg. 1977;26(4):723-726.

64. Long EG, Lawrence MC, Augustine T. ELISA for *Schistosoma mansoni* infection: durability of blood spots on filter paper. Trans R Soc Trop Med Hyg. 1981;75(5):740-741.

65. Long EG, McLaren M, Goddard MJ, Bartholomew RK, Peters P, Goodgame R. Comparison of ELISA, radioimmunoassay and stool examination for *Schistosoma mansoni* infection. Trans R Soc Trop Med Hyg. 1981;75(3):365-371.

66. McKay DA, Warren KS, Cook JA, Jordan P. Immunologic diagnosis of schistosomiasis. 3. The effects of nutritional status and infection intensity on intradermal test results in St. Lucian children. Am J Trop Med Hyg. 1973;22(2):205-210.

67. McLaren ML, Long EG, Goodgame RW, Lillywhite JE. Application of the enzyme linked immunosorbent assay (ELISA) for the serodiagnosis of *Schistosoma mansoni* infections in St. Lucia. Trans R Soc Trop Med Hyg. 1979;73(6):636-639.

68. Most H, Levine DI. Schistosomiasis in American tourists. JAMA. 1963;186:453-457.

69. Phillips SM, Colley DG. Immunologic aspects of host responses to schistosomiasis: resistance, immunopathology, and eosinophil involvement. Prog Allergy. 1978;24:49-182.

70. Pointier JP. The introduction of *Melanoides tuberculata* (Mollusca: Thiaridae) to the island of Saint Lucia (West Indies) and its role in the decline of *Biomphalaria glabrata*, the snail intermediate host of *Schistosoma mansoni*. Acta Trop. 1993;54(1):13-18.

71. Prentice MA. Schistosomiasis and its intermediate hosts in the Lesser Antillean islands of the Caribbean. Bull Pan Am Health Organ. 1980;14(3):258-268.

72. Prentice MA. Displacement of *Biomphalaria glabrata* by the snail *Thiara granifera* in field habitats in St. Lucia, West Indies. Ann Trop Med Parasitol. 1983;77(1):51-59.

73. Prentice MA. Field comparison of mouse immersion and cercariometry for assessing the transmission potential of water containing cercariae of *Schistosoma mansoni*. Annals of Tropical Medicine and Parasitology. 1983;78:169-172.

74. Prentice MA, Barnish G. Granule formulations of molluscicide for use in developing countries. Annals of Tropical Medicine and Parasitology. 1980;74:45-51.

75. Prentice MA, Barnish G. Snail infections following chemotherapy of *Schistosoma mansoni* in St. Lucia, West Indies. Trans R Soc Trop Med Hyg. 1981;75(5):713-714.

76. Prentice MA, Barnish G, Christie JD. An eco-phenotype of *Helisoma duryi* closely resembling *Biomphalaria glabrata*. Annals of Tropical Medicine and Parasitology. 1977;71:237-238.

77. Prentice MA, Christie JD, Barnish G. A miracidium trap for use in flowing water. Annals of Tropical Medicine and Parasitology. 1981;75:407-413.

78. Prentice MA, Jordan P, Bartholomew RK, Grist E. Reduction in transmission of *Schistosoma mansoni* by a four-year focal mollusciciding programme against *Biomphalaria glabrata* in Saint Lucia. Trans R Soc Trop Med Hyg. 1981;75(6):789-798.

79. Rosenfeld P, Jordan P. Testing of a schistosomiasis transmission model with field data. Bull Int Stat Inst. 1977;47:31-60.

80. Sandt DG. Evaluation of an overlay technique for the recovery of *Schistosoma mansoni* cercariae. Bull World Health Organ. 1972;47(1):125-127.

81. Sandt DG. Laboratory comparison of four cercaria recovery techniques. Bull World Health Organ. 1973;48(1):35-40.

82. Sandt DG. Direct filtration for recovery of *Schistosoma mansoni* cercariae in the field. Bull World Health Organ. 1973;48(1):27-34.

83. Sher A, Butterworth AE, Colley DG, Cook JA, Freeman GL, Jr., Jordan P. Immune responses during human schistosomiasis mansoni. II. Occurrence of eosinophil-dependent cytotoxic antibodies in relation to intensity and duration of infection. Am J Trop Med Hyg. 1977;26(5 Pt 1):909-916.

84. Sturrock BM, Sturrock RF. Laboratory studies of the host-parasite relationship of *Schistosoma mansoni* and *Biomphalaria glabrata* from St Lucia, West Indies. Ann Trop Med Parasitol. 1970;64(3):357-363.

85. Sturrock RF. An investigation of some factors influencing the survival of St. Lucian *Biomphalaria glabrata* deprived of water. Ann Trop Med Parasitol. 1970;64(3):365-371.

86. Sturrock RF. The infectivity of *Schistosoma mansoni* cercariae concentrated by a continuous flow centrifuge. Trans R Soc Trop Med Hyg. 1970;64(1):197-198.

87. Sturrock RF. The application of catalytic models to schistosomiasis in snails. J Helminthol. 1971;45(2):189-200.

88. Sturrock RF. Control of *Schistosoma mansoni* transmission: strategy for using molluscicides on St. Lucia. Int J Parasitol. 1973;3(6):795-801.

89. Sturrock RF. Field studies on the transmission of *Schistosoma mansoni* and on the bionomics of its intermediate host, *Biomphalaria glabrata,* on St. Lucia, West Indies. Int J Parasitol. 1973;3(2):175-194.

90. Sturrock RF. Field studies on the population dynamics of *Biomphalaria glabrata*, intermediate host of *Schistosoma mansoni* on the West Indian Island of St. Lucia. Int J Parasitol. 1973;3(2):165-174.

91. Sturrock RF. Problems associated with mollusciciding natural habitats. In: Cheng TC, editor. Molluscicides in Schistosomiasis Control. London: Academic Press; 1974. p. 51-65.

92. Sturrock RF. Persistence of the molluscicide Bayluscide (clonitralide) emulsifiable concentrate on mud surfaces in the tropics. Ann Trop Med Parasitol. 1974;68(4):427-434.

93. Sturrock RF. Ecological notes on habitats of the freshwater snail *Biomphalaria glabrata*, intermediate host of *Schistosoma mansoni*, on St. Lucia, West Indies. Caribbean Journal of Science. 1974;14:149-161.

94. Sturrock RF. Distribution of the snail *Biomphalaria glabrata*, intermediate host of *Schistosoma mansoni*, within a St Lucian field habitat. Bull World Health Organ. 1975;52(3):267-272.

95. Sturrock RF, Barnish G. The aerial application of molluscicides with special reference to schistosomiasis control. Bull World Health Organ. 1973;49(3):283-285.

96. Sturrock RF, Barnish G, Seeyave J. Field tests on the effect of three molluscicidal chemicals on bananas. Pflanzenschutz-Nachrichten Bayer. 1974;27:56-61.

97. Sturrock RF, Barnish G, Upatham ES. Snail findings from an experimental mollusciciding programme to control *Schistosoma mansoni* transmission on St. Lucia. Int J Parasitol. 1974;4(3):231-240.

98. Sturrock RF, Cohen JE, Webbe G. Catalytic curve analysis of schistosomiasis in snails. Ann Trop Med Parasitol. 1975;69:133-134.

99. Sturrock RF, Sturrock BM. Observations on some factors affecting the growth rate and fecundity of *Biomphalaria glabrata* (Say). Ann Trop Med Parasitol. 1970;64(3):349-355.

100. Sturrock RF, Sturrock BM. Observations on the susceptibility to *Schistosoma mansoni* from St. Lucia of several Caribbean strains of snails of the genus *Biomphalaria*. West Indian Med J. 1970;19(1):9-13.

101. Sturrock RF, Sturrock BM. Shell abnormalities in *Biomphalaria glabrata* infected with *Schistosoma mansoni* and their significance in field transmission studies. J Helminthol. 1971;45(2):201-210.

102. Sturrock RF, Sturrock BM. The influence of temperature on the biology of *Biomphalaria glabrata* (Say), intermediate host of *Schistosoma mansoni* on St. Lucia, West Indies. Annals of Tropical Medicine and Parasitology. 1972;66:385-390.

103. Sturrock RF, Upatham ES. An investigation of the interactions of some factors influencing the infectivity of *Schistosoma mansoni* miracidia to *Biomphalaria glabrata*. Int J Parasitol. 1973;3(1):35-41.

104. Sturrock RF, Woodstock L. The development of fluorescent antibodies in unisexual and bisexual *Schistosoma mansoni* infections in mice. Ann Trop Med Parasitol. 1973;67:425-430.

105. Todd CW, Goodgame RW, Colley DG. Immune responses during human schistosomiasis mansoni. V. Suppression of schistosome antigen-specific lymphocyte blastogenesis by adherent/phagocytic cells. J Immunol. 1979;122(4):1440-1446.

106. Todd CW, Goodgame RW, Colley DG. Immune responses during human schistosomiasis mansoni. VII. Further analysis of the interactions between patient sera and lymphocytes during in vitro blastogenesis to schistosome antigen preparations. Am J Trop Med Hyg. 1980;29(5):875-881.

107. Unrau GO. Individual household water supplies as a control measure against *Schistosoma mansoni*. A study in rural St Lucia. Bull World Health Organ. 1975;52(1):1-8.

108. Unrau GO. Water supply and schistosomiasis in St. Lucia. Progress in Water Technology. 1978;11:181-190.

109. Upatham ES. Rapidity and duration of hatching of St. Lucian *Schistosoma mansoni* eggs in outdoor habitats. J Helminthol. 1972;46(3):271-276.

110. Upatham ES. Effect of water depth on the infection of *Biomphalaria glabrata* by miracidia of St. Lucian *Schistosoma mansoni* under laboratory and field conditions. J Helminthol. 1972;46(4):317-325.

111. Upatham ES. Effects of some physico-chemical factors on the infection of *Biomphalaria glabrata* (Say) by miracidia of *Schistosoma mansoni* (Sambon) in St. Lucia, West Indies. J Helminthol. 1972;46(4):305-315.

112. Upatham ES. Exposure of caged *Biomphalaria glabrata* (Say) to investigate dispersion of miracidia of *Schistosoma mansoni* (Sambon) in outdoor habitats in St. Lucia. J Helminthol. 1972;46(4):297-306.

113. Upatham ES. Interference by unsusceptible aquatic animals with the capacity of the miracidia of *Schistosoma mansoni* (Sambon) to infect *Biomphalaria glabrata* (Say) under field-simulated conditions in St. Lucia, West Indies. J Helminthol. 1972;46(3):277-283.

114. Upatham ES. Studies on the hatching of *Schistosoma mansoni* eggs in standing-water and running-water habitats in St. Lucia, West Indies. South East Asian Journal of Tropical Medicine and Public Health. 1972;3:600-604.

115. Upatham ES. The effect of water temperature on the penetration and development of St. Lucian *Schistosoma mansoni* miracidia in local *Biomphalaria glabrata*. Southeast Asian J Trop Med Public Health. 1973;4(3):367-370.

116. Upatham ES. Location of *Biomphalaria glabrata* (Say) by miracidia of *Schistosoma mansoni* Sambon in natural standing and running waters on the West Indian Island of St. Lucia. Int J Parasitol. 1973;3(3):289-297.

117. Upatham ES. Letter: Effect of a waterfall on the infectivity of St. Lucian *Schistosoma mansoni* cercariae. Trans R Soc Trop Med Hyg. 1973;67(6):884-885.

118. Upatham ES. Dispersion of St. Lucian *Schistosoma mansoni* cercariae in natural standing and running waters determined by cercaria counts and mouse exposure. Ann Trop Med Parasitol. 1974;68(3):343-352.

119. Upatham ES. Infectivity of *Schistosoma mansoni* cercariae in natural St. Lucian habitats. Ann Trop Med Parasitol. 1974;68(2):235-236.

120. Upatham ES. Studies on the effects of cercarial concentration and length of exposure on the infection of mice by St Lucian *Schistosoma mansoni* cercariae in a natural running-water habitat. Parasitology. 1974;68(2):155-159.

121. Upatham ES. Field studies on the bionomics of the free-living stages of St. Lucian *Schistosoma mansoni*. Int J Parasitol. 1976;6(3):239-245.

122. Upatham ES, Sturrock RF. Studies on the effects of cercarial concentration and length of exposure on the infection of mice by *Schistosoma mansoni*. Parasitology. 1973;67(2):219-228.

123. Upatham ES, Sturrock RF. Field investigations on the effect of other aquatic animals on the infection of *Biomphalaria glabrata* by *Schistosoma mansoni* miracidia. J Parasitol. 1973;59(3):448-453.

124. Upatham ES, Sturrock RF. Preliminary trials against *Biomphalaria glabrata* of a new molluscicide formulation: gelatin granules containing Bayluscide wettable powder. Ann Trop Med Parasitol. 1977;71(1):85-93.

125. Upatham ES, Sturrock RF, Cook JA. Studies on the hatchability of *Schistosoma mansoni* eggs from a naturally infected human community on St Lucia, West Indies. Parasitology. 1976;73(3):253-264.

126. Walsh JA, Warren KS. Selective primary health care: an interim strategy for disease control in developing countries. Soc Sci Med Med Econ. 1980;14(2):145-163.

127. Warren KS. The immunopathogenesis of schistosomiasis: a multidisciplinary approach. Trans R Soc Trop Med Hyg. 1972;66(3):417-434.

128. Warren KS. Regulation of the prevalence and intensity of schistosomiasis in man: immunology or ecology? J Infect Dis. 1973;127(5):595-609.

129. Warren KS, Cook JA, David JR, Jordan P. Passive transfer of immunity in human schistosomiasis mansoni: effect of transfer factor on early established infections. Trans R Soc Trop Med Hyg. 1975;69(5-6):488-493.

130. Warren KS, Cook JA, Jordan P. Passive transfer of immunity in human schistosomiasis mansoni: effect of hyperimmune anti-schistosome gamma globulin on early established infections. Trans R Soc Trop Med Hyg. 1972;66(1):65-74.

131. Warren KS, Cook JA, Littell AS, Kagan IG, Jordan P. Immunologic diagnosis of schistosomiasis. II. Further studies on the sensitivity and specificity of delayed intradermal reactions. Am J Trop Med Hyg. 1973;22(2):199-204.

132. Warren KS, Kellermeyer RW, Jordan P, Littell AS, Cook JA, Kagan IG. Immunologic diagnosis of schistosomiasis. I. A controlled study of intradermal (immediate and delayed) and serologic tests in St. Lucians infected with *Schistosoma mansoni* and in uninfected St. Vincentians. Am J Trop Med Hyg. 1973;22(2):189-198.

133. Woodstock L, Cook JA, Peters PA, Warren KS. Random distribution of schistosome eggs in the feces of patients with Schistosomiasis mansoni. J Infect Dis. 1971;124(6):613-614.
